# Analysis of the Efficacy of Diet and Short-Term Probiotic Intervention on Depressive Symptoms in Patients after Bariatric Surgery: A Randomized Double-Blind Placebo Controlled Pilot Study

**DOI:** 10.3390/nu15234905

**Published:** 2023-11-24

**Authors:** Natalia Komorniak, Mariusz Kaczmarczyk, Igor Łoniewski, Alexandra Martynova-Van Kley, Armen Nalian, Michał Wroński, Krzysztof Kaseja, Bartosz Kowalewski, Marcin Folwarski, Ewa Stachowska

**Affiliations:** 1 Department of Human Nutrition and Metabolomics, Pomeranian Medical University in Szczecin, 71-460 Szczecin, Poland; natalia.komorniak@pum.edu.pl; 2 Sanprobi sp. z o.o. sp. k., Kurza Stopka 5/C, 70-535 Szczecin, Poland; mariusz.kaczmarczyk@sanprobi.pl (M.K.); sanprobi@sanprobi.pl (I.Ł.); 3 Department of Biochemical Science, Pomeranian Medical University in Szczecin, 71-460 Szczecin, Poland; 4 Department of Biology, Stephen F. Austin State University, Nacogdoches, TX 75962, USA; avankley@sfasu.edu (A.M.-V.K.); armen.nalian@gmail.com (A.N.); 5 Department of Psychiatry, Pomeranian Medical University in Szczecin, 71-460 Szczecin, Poland; michal.wronski@pum.edu.pl; 6 Department of General Surgery and Transplantation, Pomeranian Medical University in Szczecin, 70-111 Szczecin, Poland; kaseja@autograf.pl; 7 Independent Provincial Public Hospital Complex in Szczecin-Zdunowo, 70-891 Szczecin, Poland; zbizcz@pum.edu.pl; 8 Division of Clinical Nutrition and Dietetics, Medical University of Gdańsk, 80-211 Gdańsk, Poland; marcin.folwarski@gumed.edu.pl

**Keywords:** bariatric surgery, sleeve gastrectomy, roux-en-y gastric bypass, obesity, microbiota, gut–brain axis, beck scale, depression, diet, probiotics

## Abstract

(1) Background: studies have shown that some patients experience mental deterioration after bariatric surgery. (2) Methods: We examined whether the use of probiotics and improved eating habits can improve the mental health of people who suffered from mood disorders after bariatric surgery. We also analyzed patients’ mental states, eating habits and microbiota. (3) Results: Depressive symptoms were observed in 45% of 200 bariatric patients. After 5 weeks, we noted an improvement in patients’ mental functioning (reduction in BDI and HRSD), but it was not related to the probiotic used. The consumption of vegetables and whole grain cereals increased (DQI-I adequacy), the consumption of simple sugars and SFA decreased (moderation DQI-I), and the consumption of monounsaturated fatty acids increased it. In the feces of patients after RYGB, there was a significantly higher abundance of two members of the Muribaculaceae family, namely Veillonella and Roseburia, while those after SG had more Christensenellaceae R-7 group, Subdoligranulum, Oscillibacter, and UCG-005. (4) Conclusions: the noted differences in the composition of the gut microbiota (RYGB vs. SG) may be one of the determinants of the proper functioning of the gut–brain microbiota axis, although there is currently a need for further research into this topic using a larger group of patients and different probiotic doses.

## 1. Introduction

The benefits of bariatric surgery include improvements in psychosocial status, social relationships, and quality of life [1,2]. Symptoms of depression and anxiety decrease in the majority of patients 6–24 months after undergoing the bariatric procedure. However, they reappear in some patients after 36–60 months, sometimes even returning to pre-surgery levels [3]. Studies have shown that, compared to preoperative values, at 10 years after bariatric surgery (despite successful weight loss), patients’ mental statuses often deteriorate, including overall mental health, sense of control, and fear of intimacy [4]. The meta-analyses performed suggest that patients undergoing bariatric surgery have a higher risk of self-harm/suicide attempts than the control group, being matched in terms of age, gender, and BMI. The authors suggested that this may be related to various pre- and postoperative psychosocial, physiological, and medical factors [5,6].

One of the factors affecting mental state and mood is gut microbiota [7]. The diagnosis of mental disorders (including depression) is associated with the presence of dysbiosis [8]. In our previous study, we showed that bariatric surgery affects the gut microbiota, which may play an important role in the development of depressive and gastrointestinal symptoms [9]. Low fiber consumption and increased levels of fecal isobutyric acid may lead to intestinal inflammation. Alterations in the composition or functions of the microbiota in depressed patients may be related to inflammation, reduced production of short-chain fatty acids, impaired intestinal barrier integrity, and abnormalities in neurotransmitter, carbohydrate, and amino acid metabolic processes [10]. Psychobiotics are probiotics that have a beneficial effect on mental health [11]. Most systematic reviews and meta-analyses confirm the beneficial effects of psychobiotics on depressive symptoms in healthy people, as well as patients with major depressive disorder (MDD) and people with various concomitant psychiatric and somatic disorders [12,13]. The mechanism of action that drives psychobiotics is not well understood. It may be related to a reduction in blood levels of kynurenine [14,15], which may have neurotoxic and neurodegenerative effects [16]. Other mechanisms are related to urinary cortisol reduction, anti-inflammatory effects [13,17,18], or the increased production of brain-derived neurotrophic factor (BDNF), which correlates with antidepressant activity [19]. Supplementation with *Lactobacillus helveticus R0052* and *Bifidobacterium longum R0175* strains reduces the level of somatization, anxiety, and the intensity of depressive behaviors and the hostility index, as well as plasma cortisol levels [20].

The nutritional status of an organism is also important for proper psychological functioning. For post-bariatric surgery patients, an energy-dense diet with large amounts of vegetables and fruits and restricting the consumption of highly processed products, saturated fatty acids (SFA), and simple sugars is recommended. In addition, consuming an adequate amount of protein is crucial, which is estimated to be 60–80 g/day. Dairy products, eggs, fish, lean meat, and the seeds of legumes are recommended as the main sources of protein. Equally important is the consumption of complex carbohydrates that provide fiber, as well as adequate vitamin and mineral intake [21,22,23,24]. Such a diet is known to be helpful for improving the composition of intestinal microbiota [25,26].

Unfortunately, despite these recommendations, protein, vitamin, and mineral deficiencies are diagnosed in many patients after bariatric surgery. The main causes are insufficient consumption of individual food components, the development of food aversion, a lack of recommended nutritional supplementation, decreased secretion of hydrochloric acid in the stomach, bypassing a part of the intestine that allows absorption of nutrients, and gastrointestinal concomitants [27]. Also, in patients suffering from depression, the concentrations of many micronutrients in the diet are low, as are those of omega-3 fatty acids [28]. It is known that vitamin B deficiency contributes to the development of hyperhomocysteinemia (found in some patients after bariatric surgery), which correlates with the development of depression, for example, through the generation of oxidative stress, the remodeling of the extracellular matrix of the brain and endothelial dysfunction, and the disruption of the integrity of the blood–brain barrier [29]. A low intake of omega-3 fatty acids in the diet, combined with a high intake of omega-6 fatty acids (a typical ratio in the Western diet), increases the concentrations of lipopolysaccharide (LPS) and LPS-binding protein (LBP) in the blood, increases intestinal permeability and, thus, increases the risk of developing depressive disorders [28].

To the best of our current knowledge, there are few scientific studies addressing the relationship between dietary changes, intestinal barrier functioning, and worsening mental status in patients after bariatric surgery. The aim of the study was to evaluate the efficacy of a five week probiotic therapy with multistrain probiotic preparation (Sanprobi Barrier) and the introduction of a balanced diet to improve the mental performance of patients ≥6 months after bariatric surgery who suffered from mood disorders. The multi-strain probiotic used in the previous study, showed both a reduction in depressive symptoms [30,31], and favorable effect on metabolic risk factors. Additionally, it improves integrity of the gut barrier [32,33,34] and can modify the influence of microbiota on biochemical, physiological and immunological parameters related to obesity and inflammation [35].

## 2. Materials and Methods

Two hundred patients who had undergone a Roux-en-Y gastric bypass (RYGB) or a sleeve gastrectomy (SG) at least 6 months before, completed the Beck Depression Inventory (a self-rating scale to screen for depression symptoms). Patients with a score of ≥12 points were eligible for the study [36,37,38]. Of the 90 subjects who scored ≥12 points, 23 subjects had exclusion criteria (taking antibiotics and proton pump inhibitors in the six months before testing), and 29 subjects declined further participation in the project. This allowed the initiation of further studies with a group of 38 patients. During the first study visit, biological material (blood, feces) was collected, and anthropometric measurements and a questionnaire test were performed. Diet was assessed using a food diary for the previous 72 h.

Patients were randomized and double-blinded into two groups, one of which received a probiotic or placebo for a period of 5 weeks:

- Probiotic: Sanprobi Barrier (manufacturer: Sanprobi sp. z o. o. sp. k., Szczecin, Poland), consisting of *Bifidobacterium bifidum W23*, *Bifidobacterium lactis W51*, *Bifidobacterium lactis W52*, *Lactobacillus acidophilus W37*, *Levilactobacillus brevis W63*, *Lacticaseibacillus casei W56*, *Ligilactobacillus salivarius W24*, *Lactococcus lactis W19*, and *Lactococcus lactis W58*;
- Placebo: corn starch, maltodextrins, vegetable protein;
- Dosage: 4 capsules (2 × 10^9^ CFU)/day (taken with a meal (2 capsules in the morning and 2 capsules in the evening);
- The product is available commercially on the Polish market and its composition and dosage have been approved by the relevant health authorities.

**Figure 1:**
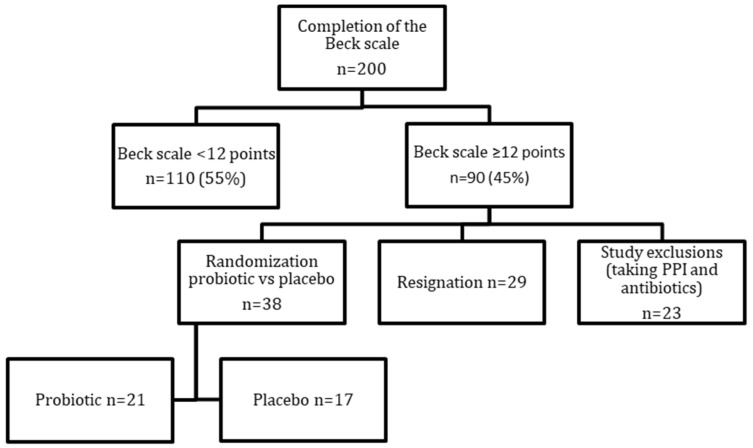
Study design. PPI—proton pump inhibitors.

Patients in both groups were educated about the principles of a healthy diet and were given a balanced meal plan tailored to the needs of patients after bariatric surgery [21]. After 5 weeks, patients were invited back for a follow-up visit, and the entire series of trials was repeated (Figure 1). All subjects were asked to bring probiotic/placebo blisters to the follow-up visit, which enabled the control of capsule taking. Additionally, a survey (food frequency questionnaire) was conducted asking about the degree of compliance with dietary recommendations and a 72 h food diary was collected.

### 2.1. Surgical Techniques

The sleeve gastrectomy was performed using the laparoscopic method. Approximately 80% of the stomach was removed along the greater curvature to form a new sleeve-like stomach, removing the fundus and body of the stomach. The remaining stomach had a capacity of about 150 mL. The Roux-en-Y Gastric Bypass was also performed laparoscopically. In this procedure, the stomach was transected creating a gastric pouch of approximately 20–30 mL, which was then anastomosed with mid-jejunum, creating the alimentary limb. The Roux-limb length was 75–150 cm [39,40,41].

### 2.2. Anthropometric Examinations

Body composition analysis was performed using a Jawon Medical ioi-353 analyzer (Jawon, Seongnam-si, Republic of Korea). Body height [cm] was measured using a metric stadiometer. Waist circumference [cm] and hip circumference [cm] were measured using a tape measure.

### 2.3. Survey Research—Mental State

The Beck Depression Inventory (BDI), a self-assessment questionnaire, was used to assess the occurrence and severity of depressive symptoms. The total score ranges from 0 to 63 points, with the higher the score, the higher the severity of depressive symptoms. ≥12 points were considered as the presence of depressive symptoms [36,37,38].

The Athenian Insomnia Scale is a scale consisting of 8 items that assess falling asleep, waking up at night and in the morning, total sleep time, sleep quality, well-being, psychophysical fitness, and daytime sleepiness. The total score ranged from 0 to 24 points, with a higher score representing poorer sleep quality. A score of ≥6 points was considered insomnia [42].

The Hamilton Depression Rating Scale (HDRS) was administered by an experienced psychiatrist. A 21-item version was used in the study, including items on guilt, suicidal ideation, circadian rhythm disturbances, agitation, anxiety, somatic symptoms, and psychomotor retardation. Responses are scored on a scale of 0–4 and 0–2. A score of ≥7 points indicates the presence of depression [43,44].

### 2.4. Eating Habits Assessment

A food frequency questionnaire (FFQ), consisting of a comprehensive list of foods and beverages with response categories indicating usual frequency of consumption over a period of time (never or almost never, once a month or less often, several times a month, several times a week, every day, several times a day) was used.

The consumption log for the last 72 h was evaluated using the Diet 5D program (Food and Nutrition Institute, Warsaw, Poland). Based on the data collected with the above tools, the International Diet Quality Index (DQI-I) was calculated, with a total score ranging from 0 to 100 points. The lower the score, the poorer the quality of the diet used. This indicator consists of four categories:

- Variety—consumption of each food group (fish, meat, eggs, legumes, vegetables, fruits, cereals) and different sources of protein (fish, meat, eggs, legumes, dairy products) (0–20 points);
- Adequacy—assessment of consumption of individual food components that need to be supplied to ensure a healthy diet and prevent malnutrition (vegetables, fruits, cereals, fiber, protein, iron, calcium, vitamin C) (0–40 points);
- Moderation—assessing the consumption of dietary components that may require a reduction in daily intake due to an increased risk of developing chronic diseases (total fat, cholesterol, saturated fatty acids, sodium, low calorie foods) (0–30 points);
- Overall dietary balance—assessment of the ratio of individual macronutrients and fatty acids (0–10 points) [45].

### 2.5. Laboratory Tests

Biological material was collected from patients at two time points—visit 1 and control (after 5 weeks). Venous blood was collected in EDTA tubes (Sarstedt, Bionovo, Legnica, Poland), centrifuged (3500 rpm for 10 min), and then the plasma and morphos fractions were separated in each eppendorf. Stool was collected using a stool sample kit (Kałszyk, Poland). The biological material was stored at −80 °C until laboratory analyses were performed.

### 2.6. Markers of the Intestinal Barrier Integrity

Fecal zonulin levels were determined by enzyme-linked immunosorbent assay (ELISA) (Immundiagnostik AG, Bensheim, Germany) according to the manufacturer’s protocol. Absorbance was measured with a spectrophotometer (Sunrise, Tecan, Männedor, Switzerland) at 450 nm.

The concentration of LPS and occludin in blood serum was determined using an ELISA assay (EIAAB SCIENCE INC, Wuhan, China), and that of LBP was determined using an ELISA assay (FineTest, Wuhan, China) according to the manufacturer’s protocols. In each case, absorbance was measured using a spectrophotometer (Sunrise, Tecan, Männedor, Switzerland) at 450 nm.

### 2.7. Sequencing Analysis of Bacterial 16S RNA Genes

DNA isolation from feces and sequencing of the V3–V4 regions of the 16S rDNA gene were performed using the Illumina MiSeq instrument (Illumina INC, San Diego, CA, USA) at the Institute of Clinical Molecular Biology, of the University of Kiel (Kiel, Germany) according to their own protocol. DNA was isolated using microcentrifuge columns with silica membrane. Extracted DNA was purified using an Agencourt AMPure^®^XP instrument (Beckman Coulter, Brea, CA, USA). Bacterial 16S RNA analysis was based on amplification of the V3–V4 regions of the 16S rRNA gene and was performed using the Metagenomic Library Construction Kit 16S (V3–V4) for Next Generation Sequencing (Takara Bio Inc., Kusatsu, Japan). Sequencing was performed using the Illumina MiSeq v3 2 × 250 bp Kit (Illumina Inc., San Diego, CA, USA).

### 2.8. Determination of Homocysteine and Vitamin D Levels in Blood Serum

The concentration of vitamin D metabolite 25 (OH) was determined by Diagnostyka SA with the automated chemiluminescence immunoassay method (CLIA) using the LIAISON XL device (DiaSorin, Vercelli, Italy). Serum homocysteine concentration was determined by ELISA (Bioassay Technology Laboratory, Shanghai, China) according to the manufacturer’s protocol. Absorbance was measured using a spectrophotometer (Sunrise, Tecan, Männedor, Switzerland) at 450 nm.

### 2.9. 16S rRNA Sequence Preprocessing

The sequencing was carried out using Illumina paired-end technology, specifically targeting the V3–V4 region of the 16S rRNA gene. To assess the quality of the raw sequencing data, a thorough initial quality screening was conducted using FastQC (version 0.12.1) and MultiQC (version 1.12) tools. Subsequently, all data preprocessing was executed using the QIIME 2 software [46]. We used the Divisive Amplicon Denoising Algorithm 2 (DADA2) [47], which is a widely used tool for denoising and quality filtering of amplicon-based sequencing data, such as 16S rRNA gene sequences. DADA2 employs a denoising algorithm to identify and correct errors within the sequencing reads. It models the error rate using the quality scores and uses this information to distinguish true biological variants (amplicon sequence variants, ASVs) from sequencing errors. After quality filtering, trimming, denoising, and chimera removal, DADA2 assigned 5685 unique ASV to each high-quality sequence and provided estimates of the abundance of each ASV within each sample (including a minimum frequency of 22.0, a 1st quartile at 23,939, a median frequency of 33,121.0, a 3rd quartile at 40,242.5, a maximum frequency of 78,764.0, and an overall mean frequency of 31,861.6). Three samples with the lowest abundance (22, 31, 43) were removed from downstream analysis. Furthermore, taxonomic classification was performed utilizing the QIIME2 implementation of VSEARCH in conjunction with the Silva 138–99 reference dataset of 16S rRNA gene sequences for taxonomic assignment. Prior to classification, an additional step involved filtering low-abundance features—the ASVs with a frequency (total abundance across samples) below 10 were excluded, allowing for a focus on robust and abundant sequences. ASVs assigned to Eukaryota, Archaea, mitochondria, and chloroplasts were excluded from the dataset due to their likely representation of contaminants or non-bacterial sequences. Lastly, the remaining ASVs were collapsed into six taxonomic levels, ranging from genus to phylum, to simplify the dataset for subsequent microbial community analysis, providing a comprehensive and refined dataset for downstream analysis.

### 2.10. Statistical Analysis

To compare baseline characteristics of patients, we utilized either the Wilcoxon test or Fisher test. To evaluate differences between treatment groups (Placebo versus Probiotic) concerning changes in clinical response variables from baseline to the end of the intervention, including Beck Depression Inventory (BDI), Hamilton Psychiatric Rating Scale for Depression (HRSD), Insomnia scale, and Diet Quality Index—International (DQI-I), we employed a mixed-effect linear model implemented in the lme4 R package (version 1.1–34) [48]. To provide more accurate and reliable estimates of the standard errors and degrees of freedom for the fixed effects (and more reliable *p* values), we used the Kenward–Roger approximation, as implemented in the lmerTest R package (version 3.1-3) [49]. To elucidate how changes in predictor variables influenced the response variables, accounting for both fixed and random effects, we used marginal effects in conjunction with predictor effect plots. Marginal effects, calculated using the marginaleffects R package (version 0.14.0), provide insight into the average change in the outcome variable for a specific predictor variable while holding all other variables constant, especially in models with multiple predictors, as they allow you to isolate the effect of one predictor variable while keeping others at fixed values. Predictor effect plots are graphical representations that show how the predicted values of the response variable change as a function of one or more predictor variables.

The analysis of gut microbiome data was conducted at baseline, where alpha-diversity measures were compared between groups using a mixed-effect model (as described above), considering the type of surgery. To test for significant differences in the composition of biological communities among groups, we applied Permutational Multivariate Analysis of Variance (PERMANOVA) implemented in the vegan R package (2.6-4) using the Bray–Curtis dissimilarity metric. Principal Coordinate Analysis (PCoA) was used for reducing the dimensionality of beta-diversity and representing it in two-dimensions.

In the analysis of genus-level data (with a 10% prevalence filtering), differential abundance analysis (DAA) was performed using five different methods: ALDEx2, ANCOMBC2, WilcoxTSS, WilcoxRarefied, and LEfSe. WilcoxTSS and WilcoxRarefied applied the Wilcoxon test to relative abundance data (TSS, total sum scaling) and rarefied data WilcoxRarefied (with sampling depth = 14,193), respectively. All statistical analyses were carried out using the R software (version 4.2.3, R Core Team (2022)) [50].

## 3. Results

### 3.1. Patients’ Characteristics

The characteristics of patients are presented in Table 1. There were no significant differences between the Placebo and Probiotic groups.

### 3.2. Psychiatric Scales

**Figure 2:**
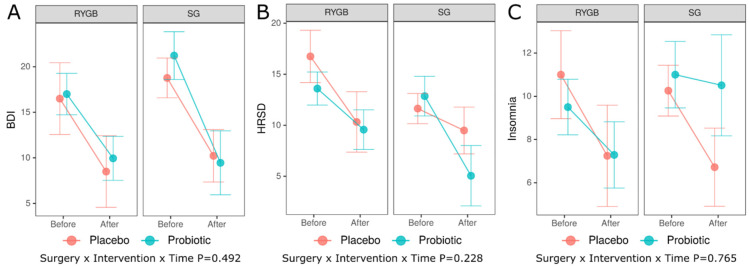
Predictor effect plots summarizing the role of intervention and surgery type on predicted values of the BDI (**A**), HRSD (**B**), and Insomnia (**C**). BDI—beck depression inventory, HRSD—Hamilton psychiatric rating scale for depression, RYGB—Roux-en-Y gastric bypass, SG—sleeve gastrectomy. P—*p* value obtained from a general mixed-effects model for the three-way interaction, i.e., type of surgery (RYGB versus SG) by intervention (Placebo versus Probiotic) by time (Before versus After); BDI—Beck Depression Inventory, HRSD—Hamilton Psychiatric Rating Scale for Depression, RYGB—Roux-en-Y Gastric Bypass, SG—Sleeve Gastrectomy.

First, we examined differences between treatments groups (Placebo versus Probiotic) with respect to the average change from baseline to end of intervention in three response variables, i.e., Beck Depression Inventory (BDI), Hamilton Psychiatric Rating Scale for Depression (HRSD), and Insomnia scale, while accounting for the type of bariatric surgery (RYGB versus SG, Figure 2).

The lack of significant interaction between the type of surgery, intervention and time (*p* = 0.492, *p* = 0.228, *p* = 0.765 for BDI, HRSD and Insomnia, respectively) indicates that the average changes from baseline to end-point were not significantly different. The differences of predicted outcomes from baseline to end-point (Before versus After)—average marginal effects (AME) evaluated for all combinations of the two remaining categorical predictors (type of surgery and intervention) are summarized in Table 2. Although the changes over time were significant in some cases, especially for BDI, pairwise comparisons of these changes between Placebo and Probiotic groups did not reveal any significant differences.

### 3.3. DQI-I and Its Subscales

**Figure 3:**
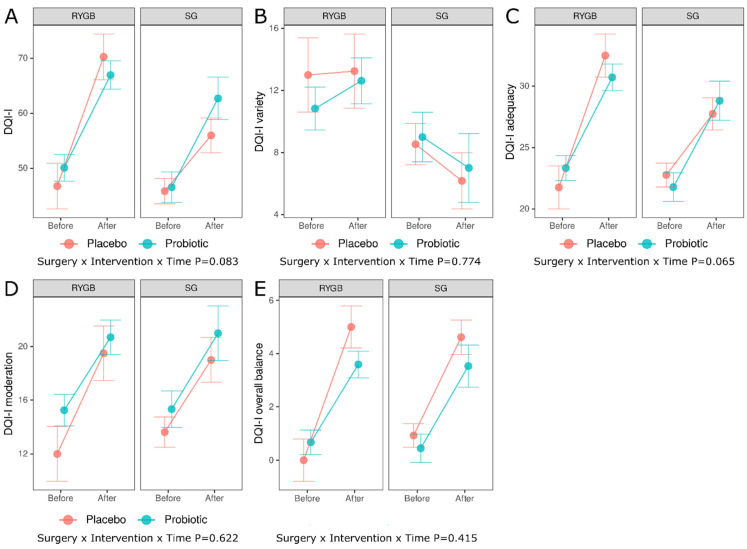
Predictor effect plots summarizing the role of intervention and surgery type on predicted values of the DQI-I (**A**), DQI-I variety (**B**), DQI-I adequacy (**C**), DQI-I moderation (**D**), and DQI-I overall balance (**E**), RYGB—Roux-en-Y Gastric Bypass, SG—Sleeve Gastrectomy, DQI-I—diet quality index international.

In addition to psychiatric outcomes, Diet Quality Index-International (DQI-I) and four subscales, namely variety, adequacy, moderation and overall balance, were also examined. Figure 3 and Table 3 shows all DQI-I related outcomes.

**Figure 4:**
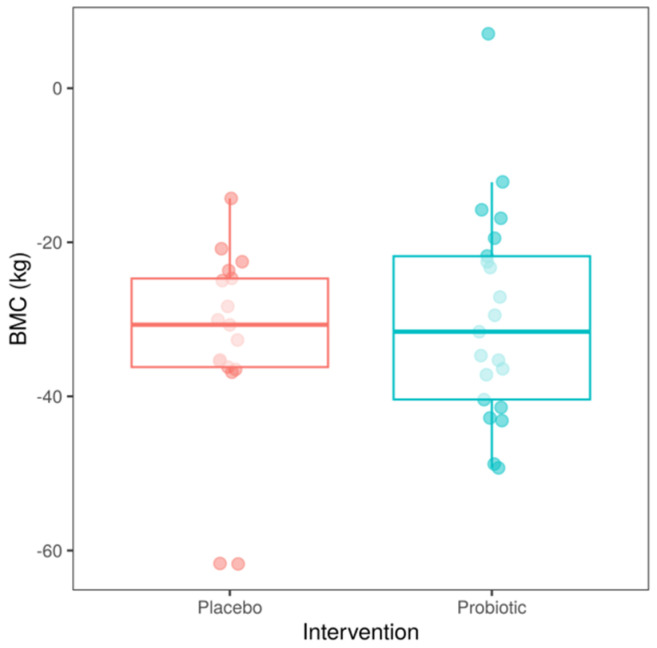
Body mass change (BMC) between the time of patient’s surgery and the start of the study.

In the next stage, we investigated the effect of body mass change (BMC), in addition to intervention, to see if patients who had experienced a higher loss of body mass between the time of their surgery and the start of the trial had a distinct reaction to the probiotic intervention. BMC in patients receiving placebo and probiotic is shown in Figure 4. In subsequent analyses, we excluded three patients with extreme values (outliers, i.e., −61.7, −61.7 and 7.1 kg). 

The BMC was computed as the difference between the patient’s body mass at the start of the study and the patient’s body mass at the time of surgery; hence, a decrease is indicated by a negative number, while an increase is indicated by a positive value; BMC did not differ between groups, *p* = 0.837 (Wilcoxon test).

### 3.4. Gut Microbiota

**Figure 5:**
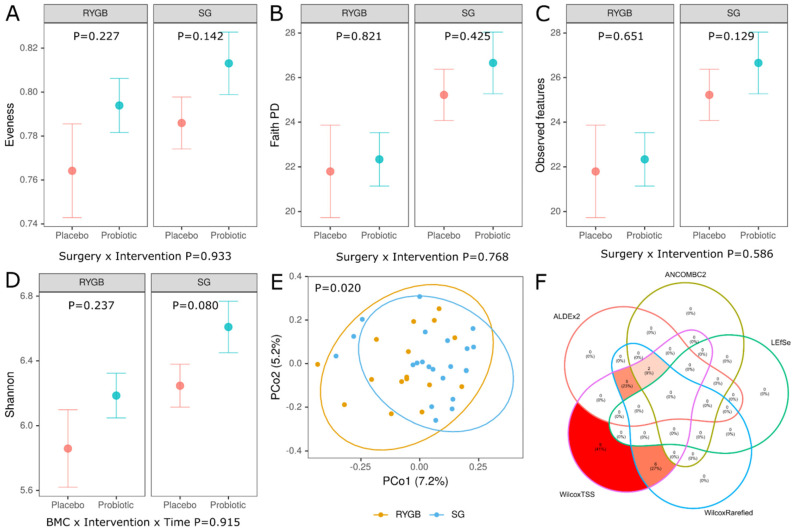
Alpha-diversity, beta-diversity and differential abundance analysis at baseline. Alpha-diversity (**A**–**D**), beta-diversity using Bray–Curtis distance (**E**) and Venn diagram (**F**). Below plots in (**A**–**D**), *p* values are obtained from a general mixed-effects model for the two-way interaction, i.e., type of surgery (RYGB versus SG) by intervention (Placebo versus Probiotic) while *p* values in each facet refer to Placebo versus Probiotic comparison in the RYGB and SG group; (**E**)—Principal-coordinate analysis (PCoA) ordination plot based on Bray–Curtis distance metrics demonstrating significant grouping of samples (PERMANOVA F = 1.27, *p* = 0.020 RYGB versus SG); (**F**)—Venn diagram comparing 5 methods of differential abundance analysis at the genus level (10% prevalence filtered) (ALDEx2, ANCOMBC2, WilcoxTSS, WilcoxRarefied and LEfSe) [51,52,53], WilcoxTSS and WilcoxRarefied use Wilcoxon test on relative abundance data (TSS, total sum scaling) or rarefied data (sampling depth = 14,193), RYGB—Roux-en-Y Gastric Bypass, SG—Sleeve Gastrectomy.

At baseline, alpha diversity did not differ between Placebo and Probiotic groups regardless of the type of bariatric surgery (Figure 5A–D). In contrast, we found a significant difference (PERMANOVA *p* = 0.020) in overall taxonomic composition between RYGB versus SG (but not between Placebo and Probiotic, PERMANOVA *p* = 0.777), as demonstrated in PCoA of Bray–Curtis distances (Figure 5E) which shows a separation between RYGB and SG. In line with this finding, there were numerous differences in the abundance of individual taxa between these groups and the results of differential abundance analysis (DAA) at the genus level using various methods are shown in Figure 5F. The Venn diagram illustrates a correspondence of 5 DAA methods and reveals that 7 genera, genus belonging to Muribaculaceae family (enriched in the RYGB), Christensenellaceae R-7 group (enriched in the SG), Veillonella (enriched in the RYGB), Subdoligranulum (enriched in the SG), Roseburia (enriched in the RYGB), Oscillibacter (enriched in the SG) and UCG-005 (enriched in the SG), were identified as differentially abundant between RYGB and SG in the ALDEx2, WilcoxTSS and Wilcox. When ANCOMBC2 method was considered, only two of them (genus belonging to Muribaculaceae family, Christensenellaceae R-7 group) remained differentially abundant. The taxa at higher levels of taxonomy were also found to be differentially abundant (Figure S1).

## 4. Discussion

To the best of our knowledge, the study conducted is the first to address the possibility of reducing the severity of depressive disorders in people after bariatric surgery using diet and probiotic therapy.

Depressive symptoms were observed in 45% of 200 patients who underwent bariatric surgery. The presence of depressive disorders was also noted in other studies. Alsubaie et al. [54] showed that 30.4% of patients suffered from moderate or severe depression and 33% from moderate or severe anxiety disorders after bariatric surgery. In addition, the authors associated the occurrence of depression with young age, postoperative complications, and psychiatric symptoms that occurred 1 to 2 years after surgery [54]. It seems that disturbances in the function of the cerebral and intestinal axes may also have a significant influence on the deterioration of the mental state of patients after bariatric surgery [55].

At present, there are lack of data in the literature examining the relationship between changes in the microbiota and the development of depression after bariatric surgery. Finding such marker bacteria in the future could be a very valuable predictor of the development of depression. In a previous paper, we published results that indicate a role for the gut microbiota and inflammatory processes in the development of depressive disorders in patients after bariatric surgery [9]. Of note, the bariatric surgery itself significantly affects the composition of the microbiota [56]. In our study, we noted significant differences in the composition of the gut microbiota of patients undergoing RYGB and SG surgery. In the feces of patients after RYGB, there was a significantly higher abundance of Muribaculaceae family, Veillonella and Roseburia, while those after SG had more Christensenellaceae R-7 group, Subdoligranulum, Oscillibacter and UCG-005. Muribaculaceae produce enzymes that break down complex carbohydrates. In one study conducted in an animal model, the authors suggested that their higher abundance may be related to higher dietary fiber intake [57,58]. Although in another study, Muribaculaceae appear to be positively correlated with body weight and have been linked to the lean phenotype [59]. Interestingly, their numbers are also positively related to depressive and anxiety behaviors [60]. In contrast, the relative abundance of Veillonella was lower in studies involving participants with anxiety and depression [61]. Another study [62] found an association of thirteen microbial taxa, including genera Eggerthella, Subdoligranulum, Coprococcus, Sellimonas, Lachnoclostridium, Hungatella, Ruminococcaceae (UCG002, UCG003 and UCG005), LachnospiraceaeUCG001, Eubacterium ventriosum and Ruminococcusgauvreauiigroup, and family Ruminococcaceae with depressive symptoms. These bacteria are known to be involved in the synthesis of glutamate, butyrate, serotonin and gamma amino butyric acid (GABA), which are key neurotransmitters for depression. In addition, the number of *Clostridiales* also seems to be important for emotional functioning [63]. The number of *Clostridiales* (including *Faecalibacterium*, *Roseburia*, *Lachnospira*, *Anaerostipes*) is reduced in many mental disorders, which is associated with disturbances in amino acid and carbohydrate metabolism [63]. It is worth noting that *Roseburia* is one of the most important butyrate-producing bacteria, which is important for modulating the brain–gut axis [64,65]. The presence of *Roseburia* has been associated with good cognitive abilities [63,66] and increased insulin sensitivity [67], while insulin resistance is associated with high systemic branched-chain amino acid concentrations [68]. Excess consumption of valine, leucine, and isoleucine may lead to a decrease in brain tryptophan (a precursor of serotonin) concentration and thus to a decrease in brain serotonin concentration [69,70]. When the excess of branched-chain amino acids competes with tryptophan, the transport of this amino acid across the blood–brain barrier is less efficient [70,71]. The authors of one of the papers [63] suggested that the gut microbiota contribute (at least indirectly) to occurrence of mental disorders through low numbers of *Clostridiales*. This bacterial order, very important for the homeostasis of the organism, plays a significant role in the degradation of branched amino acids and prevents their increased concentration in the bloodstream (which could counteract the decrease in serotonin synthesis in the central nervous system) [63].

In our study, after 5 weeks, we noted an improvement in patients’ mental functioning (reduction in BDI and HRSD), but it was not related to the probiotic used (Figure 2). Effect of probiotics after bariatric surgery does not seem very significant. A systematic review by Cook et al. [72] also found no effect of probiotics on quality of life and weight loss after bariatric surgery. This may be due to significant changes in the composition of the microbiota after surgery, functional changes (e.g., food passage) of gastrointestinal tract, changes in body weight and dietary habits. The dosage, method and timing of probiotic use are adapted to the anatomically and functionally typical gastrointestinal tract. There are no data on the adhesion and other mechanisms of action of probiotics in the gastrointestinal tract undergoing surgery.

In our study, the most important factor that contributed to the reduction in the severity of depressive disorders may have been the dietary intervention. Consumption of vegetables and whole grain cereals increased (DQI-I adequacy), consumption of simple sugars and SFA decreased (moderation DQI-I), and consumption of monounsaturated fatty acids derived mainly from olive oil increased (overall balance DQI-I). A Western-style diet, characterized by the consumption of highly processed products with a high content of sugars and saturated fatty acids and, at the same time, a low consumption of vegetables, fruits and fiber, favors the development of microbiota disorders [73]. This type of diet predisposes to low microbiological diversity, increased intestinal barrier permeability, endotoxemia, and chronic low-intensity inflammation, which can contribute to the development of many diseases [7,73]. In addition, a diet with a high glycemic load increases the risk of developing mood disorders, fatigue, and severity of depression symptoms compared with diets based on a low glycemic load (especially in overweight or obese people) [74]. On the other hand, a diet rich in fruits, vegetables, nuts, legumes, and whole grains seems to have a beneficial effect on psychophysical performance [75]. This type of diet has been shown to be associated with a lower risk of MDD and anxiety disorders than in people following the Western diet [76]. In addition, the introduction of a Mediterranean diet has been associated with an improvement in depression symptoms in patients suffering from MDD with low diet quality [77].

The original composition of the microbiota may be important for the functioning of the intestinal barrier and the effectiveness of its sealing. It seems that RYGB has a greater impact on the gut microbiota than restrictive surgery [72]. This could be due to the fact that the anatomical changes induced by the RYGB procedure reduce the absorptive surface of the intestine and also result in the stomach being exposed to higher amounts of gastric acid. In addition, the passage of food through the gastrointestinal tract is accelerated, and the increased pH in the intestine alters its redox potential, which affects the increase in the number of aerobic and facultative aerobic microorganisms, i.e., *Proteobecteria* [78].

In our study, we noted no significant differences in parameters assessing intestinal barrier integrity (LPS, LBP, zonulin, occludin) between the group taking the probiotic and the placebo. However, the study by Clemente-Postigo et al. [75] showed that the concentration of LPS and LBP in the blood depended on the bariatric surgery performed and the previous blood glucose level of the patient. LPS and LBP concentrations decreased significantly (compared with preoperative values) on the ninetieth day after SG surgery. In contrast, LBP levels increased on the 15th day after bile duct exclusion and returned to preoperative levels on the 90th day after surgery. The values increased again on the 90th day after surgery [79]. In turn, Yang et al. [80] showed that LBP levels were related to BMI and high-sensitivity C-reactive protein, and that LBP levels decreased significantly one year after bariatric surgery compared with preoperative levels. The exact mechanisms leading to changes in LPS and LBP concentrations in the blood of patients after bariatric surgery are not known. They appear to be related to changes in gut microbiota composition, diet, and severity of inflammation [80,81].

Due to significant limitations, this study should be considered a pilot study. Small study groups, short time of intervention, high heterogeneity and difficulties in compliance were the biggest limitations. However, the topic addressed is very interesting and requires further well-designed clinical and mechanistic studies.

## 5. Conclusions

In our study, we noted a reduction in the severity of depressive symptoms, although it was not related to the probiotic therapy used. Of note, in patients who have undergone bariatric surgery, special attention should be paid to the proper balance of meals (as one of the elements contributing to the maintenance of mental health). In addition, the noted differences in the composition of the gut microbiota (RYGB vs. SG) may be one of the determinants of the functioning of the gut–brain microbiota axis, although there is currently a need for further research on this topic with a larger group of patients and different probiotic doses.

## Figures and Tables

**Table 1:** Characteristics of patients.

	Placebo		Probiotic		*P*	Q
	*n*	Mean ± sd, *n* (%)	*n*	Mean ± sd, *n* (%)		
age	17	44.4 ± 10.4	21	44.9 ± 10.7	0.659	0.843
Beck scale	17	18.2 ± 7.1	21	18.8 ± 8.4	0.952	0.971
Hamilton scale	16	12.9 ± 4.6	17	13.3 ± 4.7	0.971	0.971
Insomnia scale	16	10.5 ± 4.8	17	10.1 ± 3.1	0.612	0.843
Waist circumference (cm)	17	100.6 ± 11.4	21	96.2 ± 12.7	0.277	0.665
WHR	17	0.86 ± 0.07	21	0.85 ± 0.09	0.702	0.843
Weight (kg)	17	93.2 ± 18.8	21	84.8 ± 15.5	0.168	0.589
FFM (kg)	17	59.3 ± 10.0	21	57.1 ± 10.4	0.463	0.788
Fat mass (kg)	17	33.9 + 11.5	21	29.1 ± 8.2	0.191	0.589
BMI (kg/m^2^)	17	32.2 ± 5.3	21	30.1 ± 4.5	0.127	0.589
Weight at surgery day (kg)	17	125.9 ± 24.2	21	114.5 ± 16.0	0.167	0.589
Time after surgery (months)	17	41.3 ± 41.8	21	28.4 ± 27.4	0.462	0.788
LBP (ng/mL)	17	551 ± 127	21	643 ± 236	0.127	0.589
LPS (pg/mL)	17	107.9 ± 33.4	21	97.9 ± 36.6	0.252	0.665
Homocysteine (nmol/mL)	17	8.4 ± 10.4	21	8.6 ± 8.8	0.411	0.788
Zonulin (ng/mL)	14	145.7 ± 84.6	15	128.9 ± 63.4	0.777	0.847
Occludin (ng/mL)	17	13.2 ± 3.4	21	14.1 ± 3.6	0.500	0.788
Vitamin D (ng/mL)	17	19.4 ±7.9	19	20.3 ± 6.5	0.318	0.694
DQI_I (points)	17	46.1 ± 9.7	21	48.6 ± 7.5	0.186	0.589
Variety_DQI_I (points)	17	9.6 ± 4.8	21	10.0 ± 4.9	0.677	0.843
Adequacy_DQI_I (points)	17	22.5 ± 3.6	21	22.7 ± 3.5	0.746	0.847
Moderation_DQI_I (points)	17	13.2 ± 4.8	21	15.3 ± 4.3	0.196	0.589
Overall_balance_DQI_I (points)	17	0.71 ± 1.99	21	0.57 ± 0.93	0.196	0.788
Surgery type (RYGB/SG)	17	4 (23.5)/13 (76.5)	21	12 (57.1)/9 (42.9)	0.052	0.589

sd—standard deviation, WHR—waist-to-hip ratio, FFM—fat-free mass, BMI—body mass index, LBP—lipopolysaccharide binding protein, LPS—lipopolysaccharide, DQI-I—diet quality index international, RYGB—Roux-en-Y gastric bypass, SG—sleeve gastrectomy.

**Table 2:** Average marginal effects as a difference in predicted outcomes (Before versus After) for all combinations of levels of the categorical predictor (type of surgery and intervention) for psychiatric outcomes.

Outcome	Surgery	Intervention	Est	SE	*p*	Est_pairwise_	SE_pairwise_	P_pairwise_
BDI	RYGB	Placebo	−8.00	3.49	0.022	−0.95	4.11	0.817
		Probiotic	−7.05	2.17	0.001			
	SG	Placebo	−8.55	2.69	0.002	3.22	4.25	0.449
		Probiotic	−11.76	3.29	<0.001			
HRSD	RYGB	Placebo	−6.43	3.86	0.096	−2.40	4.59	0.601
		Probiotic	−4.03	2.49	0.106			
	SG	Placebo	−2.15	2.71	0.428	5.66	4.42	0.201
		Probiotic	−7.80	3.50	0.026			
Insomnia	RYGB	Placebo	−3.76	2.92	0.199	−1.54	3.48	0.658
		Probiotic	−2.21	1.89	0.242			
	SG	Placebo	−3.54	2.09	0.090	−3.05	3.40	0.370
		Probiotic	−0.49	2.68	0.855			

Examined contrasts in all cases—Before versus After (After minus Before), BDI—Beck Depression Inventory, HRSD—Hamilton Psychiatric Rating Scale for Depression, RYGB—Roux-en-Y Gastric Bypass, SG—Sleeve Gastrectomy, Est—estimate of average marginal effect (AME), SE—standard error, *p*—*p* value for testing the significance of AME estimates, Est_pairwise_, SE_pairwise_, P_pairwise_—estimates of AME, standard error and *p* value, respectively, for pairwise comparisons between Placebo and Probiotic group (Placebo minus Probiotic).

**Table 3:** Average marginal effects as a difference in predicted outcomes (Before versus After) for all combinations of levels of the categorical predictor (type of surgery and intervention) for DQI-I.

Outcome	Surgery	Intervention	Est	SE	*p*	Est_pairwise_	SE_pairwise_	P_pairwise_
DQI-I	RYGB	Placebo	23.5	4.12	<0.001	6.64	4.85	0.171
		Probiotic	16.9	2.55	<0.001			
	SG	Placebo	10.1	3.14	0.001	−6.02	4.95	0.224
		Probiotic	16.1	3.83	<0.001			
DQI-I variety	RYGB	Placebo	0.25	2.38	0.916	−1.54	2.80	0.582
		Probiotic	1.79	1.47	0.224			
	SG	Placebo	−2.36	1.81	0.192	−0.37	2.86	0.897
		Probiotic	−1.99	2.21	0.268			
DQI-I adequacy	RYGB	Placebo	10.75	1.64	<0.001	3.37	1.94	0.082
		Probiotic	7.39	1.02	<0.001			
	SG	Placebo	4.98	1.26	<0.001	−2.06	1.99	0.300
		Probiotic	7.04	1.54	<0.001			
DQI-I moderation	RYGB	Placebo	7.50	2.89	0.009	2.05	3.38	0.544
		Probiotic	5.45	1.75	0.002			
	SG	Placebo	5.38	2.02	0.008	−0.28	3.18	0.929
		Probiotic	5.67	2.46	0.021			
DQI-I overall balance	RYGB	Placebo	5.00	1.09	<0.001	2.08	1.27	0.103
		Probiotic	2.92	0.66	<0.001			
	SG	Placebo	3.69	0.77	<0.001	0.61	1.21	0.614
		Probiotic	3.08	0.94	<0.001			

RYGB—Roux-en-Y Gastric Bypass, SG—Sleeve Gastrectomy, DQI-I—diet quality index international, Est—estimate of average marginal effect (AME), SE—standard error, *p*—*p* value for testing the significance of AME estimates, Est_pairwise_, SE_pairwise_, P_pairwise_—estimates of AME, standard error and *p* value, respectively, for pairwise comparisons between Placebo and Probiotic group (Placebo minus Probiotic).

## Data Availability

Data are contained within the article and Supplementary Materials.

## Supplementary Materials

The following supporting information can be downloaded at: https://www.mdpi.com/article/10.3390/nu15234905/s1, Figure S1: Gut microbiota—the different abundance of taxa at higher levels of taxonomy.
